# Activation of nuclear factor-kappa B by TNF promotes nucleus pulposus mineralization through inhibition of ANKH and ENPP1

**DOI:** 10.1038/s41598-021-87665-2

**Published:** 2021-04-15

**Authors:** Agata K. Krzyzanowska, Robert J. Frawley, Sheela Damle, Tony Chen, Miguel Otero, Matthew E. Cunningham

**Affiliations:** 1 HSS Research Institute, Hospital for Special Surgery, 515 E 71st Street, New York, NY 10021 USA; 2 Weill Cornell Graduate School of Medical Sciences, 1300 York Avenue, LC501, New York, NY 10065 USA; 3 Weill Cornell Medical College, New York City, 1300 York Avenue, LC501, New York, NY 10065 USA; 4 Hospital for Special Surgery, 535 E 70th Street, New York, NY 10021 USA

**Keywords:** Translational research, Spine structure

## Abstract

Spontaneous mineralization of the nucleus pulposus (NP) has been observed in cases of intervertebral disc degeneration (IDD). Inflammatory cytokines have been implicated in mineralization of multiple tissues through their modulation of expression of factors that enable or inhibit mineralization, including TNAP, ANKH or ENPP1. This study examines the underlying factors leading to NP mineralization, focusing on the contribution of the inflammatory cytokine, TNF, to this pathologic event. We show that human and bovine primary NP cells express high levels of ANKH and ENPP1, and low or undetectable levels of TNAP. Bovine NPs transduced to express TNAP were capable of matrix mineralization, which was further enhanced by ANKH knockdown. TNF treatment or overexpression promoted a greater increase in mineralization of TNAP-expressing cells by downregulating the expression of ANKH and ENPP1 via NF-κB activation. The increased mineralization was accompanied by phenotypic changes that resemble chondrocyte hypertrophy, including increased RUNX2 and COL10A1 mRNA; mirroring the cellular alterations typical of samples from IDD patients. Disc organ explants injected with TNAP/TNF- or TNAP/shANKH-overexpressing cells showed increased mineral content inside the NP. Together, our results confirm interactions between TNF and downstream regulators of matrix mineralization in NP cells, providing evidence to suggest their participation in NP calcification during IDD.

## Introduction

The nucleus pulposus (NP) is the hydrated central gelatinous tissue of the intervertebral disc (IVD), essential for the appropriate distribution of load throughout the spine. NP is avascular and not calcified in healthy, physiological conditions, but calcification of the NP is observed in pathological states such as IVD degeneration (IDD)^1,2^. Disc calcification is common in the thoracic spine (up to 80% in older patients), typically involving the lower thoracic levels, correlating with disc height loss and is more common in men, but does not correlate with vacuum phenomenon or endplate abnormalities^3^. Lumbar IVD calcification is much less common, and has been reported to be composed of calcium pyrophosphate dihydrate deposits (in 3% of examined spines), as hydroxyapatite (HA, 12%), or as a hydroxyapatite-like (19%, with similar roentgenographic appearance but non-identical X-ray diffraction as HA), and to be correlated with degenerative findings such as IVD height loss, endplate disruption, vacuum phenomenon and osteophytes^4^. In patients undergoing lumbar discectomy for disc herniations in the setting of IDD, as compared to normal cadaveric IVD tissue, disc calcification and evidence of angiogenesis were more prevalent in the IDD samples^5^. Although not well described, there may be different factors controlling IVD calcification in the thoracic and lumbar regions of the spine, where thoracic IVD calcification does not require as much degeneration as observed with lumbar IVD calcification, either due to differing calcium crystals being deposited by segment, differences in the mechanics and propensity for degeneration of these spine segments, or differences in the cells within the IVDs responding to the matrix degeneration and applied stresses.

Our understanding of tissue mineralization in general has grown dramatically in recent years. Matrix mineralization is governed by the balance of extracellular inorganic phosphate (ePi) and extracellular inorganic pyrophosphate (ePPi)^6^. ePPi is a potent inhibitor of hydroxyapatite deposition^7^, and ePi is a necessary substrate for bone mineral formation^8^. Progressive ankylosis protein homolog (ANKH)^9^ and ectonucleotide pyrophosphatase/phosphodiesterase (ENPP1)^10^ are both important upregulators of ePPi, are generally expressed in all tissues^11^, and in murine knockout models predispose to generalized joint arthrosis and ankylosis due to profuse pathological mineral deposition. Conversely, tissue nonspecific alkaline phosphatase (TNAP) promotes tissue mineralization by hydrolysis of ePPi and creation of ePi^7,8^, has a required expression in mineralized tissues^11^, and when deficient in murine models predisposes to poor bone mineralization and impaired growth among other deficiencies^12^. Accordingly, enforced TNAP expression in normally non-mineralized tissues causes pathological mineralization^11^, and co-knockdown of ANKH/TNAP^13^ or ENPP1/TNAP^14^ expression normalizes murine pathological mineralization phenotypes, emphasizing the importance of the balance of these gene regulators in modulating the ePi/ePPi ratio.

Regulation of mineralization has more than genetic constraints. ANKH knockout mice and ENPP1 deficient animals demonstrate mineral deposition in the annulus fibrosus (AF) that is more exuberant in the ANKH/ENPP1 double knockout, but no mineral is noted in the NP^13^. Proliferated hypertrophic chondrocyte-like cells distinct from the expected fibrocartilagenous AF cells were noted in the ENPP1 deficient animals, along with abnormal expression of matrix Gla protein^15^, whereas proliferated hyaline-like cartilage and foci of necrosis were noted in the discs of ANKH knockout animals^16^, but neither mutation produced mineralization in the NP portion of the discs, suggesting simple control of ePi/ePPi ratio was not the entire story. In IDD patients, inflammatory cytokines such as tumor necrosis factor (TNF) or interleukin (IL)-1β are highly expressed in recovered disc tissue and experimentally these cytokines have been shown to contribute to disc pathology, extracellular matrix degradation, and changes in cell phenotype^17–20^. TNF down-regulates ANKH gene expression via canonical p65 NF-κB signaling in human aortic smooth muscle cells (hASMC), leading to decreased ePPi and vascular calcification^21^. Further, both IL-1β and TNF strongly induced deposition of hydroxyapatite in bone marrow-derived human mesenchymal stem cells (hMSCs) in vitro, by both up-regulating TNAP activity^22^, and by IL-1β decreasing ENPP1 activity^23^, thereby raising the ePi/ePPi ratio. Similarly, a down-regulation of ANKH was observed in NP cells treated with IL-1β in vitro^24,25^, and in bovine NP (bNP) cells gene-programmed to overexpress TNAP, IL-1β was shown to induce matrix mineralization^25^. Together, these results support the notion that inflammatory cytokines could contribute to the pathologic tissue mineralization observed in IDD, by differentially modulating the expression of the ANK/ENPP/TNAP genes in NP cells. Additionally, it should be noted that mineralization may be further enhanced by other unrecognized mechanisms co-activated by the cytokine signaling.

While disc mineralization represents a pathological state associated to IDD, mineralization and bone production within the disc is a desired result during spinal fusion surgery, a procedure where two or more adjacent vertebrae are permanently connected together to eliminate painful motion, relieve back pain, realign spinal imbalance, or restore spinal stability^26^. Failure to induce sufficient bone to stably fuse the intended vertebrae is a vexing problem in spine surgery, and particular to anterior spine fusions may be related to retained/persistent NP in the IVD as suggested from fusion results in comparative models^27,28^, leading at least several authors to investigate and suggest that there are inhibitory signals expressed by NP cells that inhibit osteogenesis^29–32^. Bone Morphogenetic Proteins (BMPs) are members of the TGF-β superfamily, are FDA approved for clinical use for 1–2 level anterior IVD spine fusions with cages, and have been shown to be remarkably successful in augmenting fusion success when delivered to well prepared intended fusion sites^33,34^. Interestingly, in comparative models where *intact* discs are injected with TGF-β family members in the form of factors^35,36^ or implanted with gene-therapies to express factors^37–39^, overall disc health is maintained (or improved in needle puncture IDD models)^32,40^, but when these same factors are implanted in IVDs where more significant *trauma* has occurred, bone can be generated and fusions obtained in vivo^41,42^ or ex vivo^43^, but not in a predictable or reproducible manner. Riew et al.^44^ reported very favorable results for anterior IVD fusion in pigs following thoracoscopic NP removal and implantation of autologous MSCs adenovirus infected to express BMP-2, a minimally invasive technique, but otherwise similar to the NP debridement performed in clinical anterior spine fusions. Culture treatment^29,45^ or gene-therapy^46^ methods have been used to assess if NP cells become more bone-like with various pro-mineralization treatments, with varied success as regards induction of bone-like genes or NPs capable of matrix mineral production, but it is less convincing that the NPs were transformed into bone-like cells capable of robust osteogenesis. The one exception to this is co-culture of human NPs (hNPs) with human mesenchymal stromal cells treated with L51P, a BMP2 mutant that is deficient in BMP-RI binding and has been shown to suppress noggin function^47^. L51P neutralizes hNP inhibition of the stromal cells, rescuing their ability to mineralize their matrix and gene regulate RunX2, collagen-1, and osteopontin at near normal levels^48^. Although L51P binds and inactivates noggin, there must be an additional signaling influence, possibly through BMP-RII binding and signaling or through signaling mediated by the L51P-noggin complex, because if the only effect of L51P was to increase BMP levels, it would be expected to make the NP cells be more inhibitory, robust, healthy and disc-like, as described above. Clinical IVD fusions currently depend on surgical intervention; however, improving our understanding of the pathways involved in disc mineral homeostasis, IDD, and ectopic mineralization could lead to the development of novel, efficacious and less invasive therapeutic approaches aimed to induce disc matrix mineralization and bone production in the NP of the IVD, resulting in spinal fusion.

In this study, we investigated the molecular mechanisms by which inflammatory cytokines drive tissue mineralization in NP cells of the IVD in order to apply this knowledge to develop a well-controlled and non-invasive means of inducing NP mineralization. Specifically, we aimed to (1) characterize the contribution of TNF in the regulation of TNAP, ANKH and ENPP1 in NP cells in vitro*,* and show how relative expression of these genes alters the ability of NP cells to mineralize their matrix, and (2) translate these findings into an ex vivo organ culture model of IVD mineralization, with the goal to induce mineral formation within the IVD in a targeted and controlled fashion. We hypothesized that NP cells would express ENPP/ANKH but not TNAP and they would not vigorously mineralize their extracellular matrix, that NP cells gene-programmed to express TNAP would gain the ability to mineralize their matrix, that suppressing ENPP/ANKH in TNAP expressing NP cells would further strengthen their matrix mineralizing ability, and that NP cells gene-programmed to vigorously mineralize their extracellular matrix in vitro would be capable of intra-discal matrix mineralization when injected into intact disc organs ex vivo. NP cell gene-programming to confer matrix mineralization capacity within the disc space may provide a stepping stone towards a novel injection-based minimally-invasive spinal fusion method.

## Results

### TNF decreases the expression of inhibitors of mineralization and accelerates mineral deposition in TNAP-expressing NP cells

**Figure 1:**
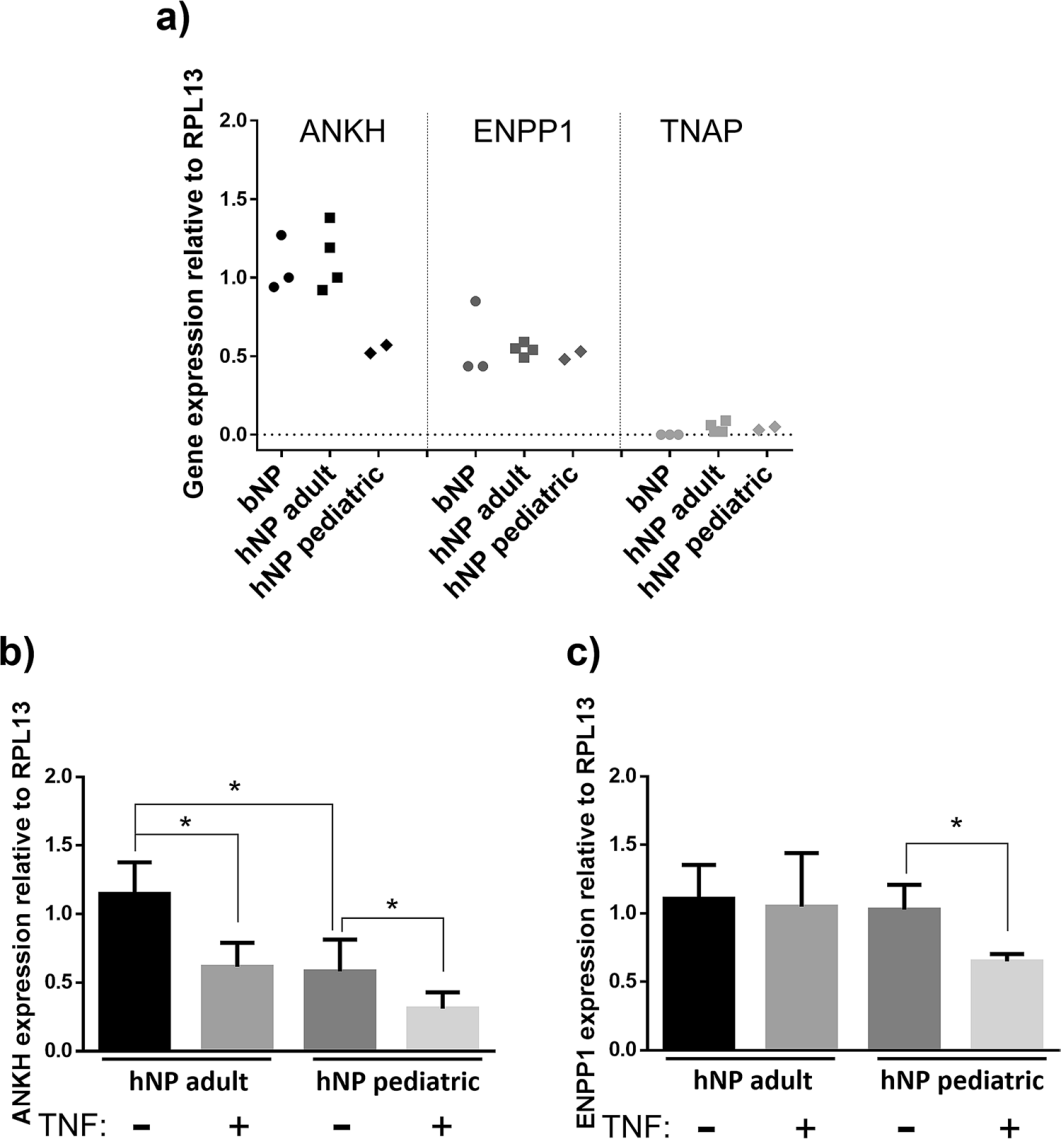
TNF down-regulates ANKH and ENPP1 expression in human NP cells. (**a**) Gene expression of ANKH, ENPP1 and TNAP in primary bNP and primary hNP cells obtained from pediatric and adult patients; (**b**) effect of 20 ng/mL TNF treatment on ANKH and (**c**) ENPP1 gene expression in hNP (n = 4). Significance represents p < 0.05. Data analyzed using one-way ANOVA.

**Figure 2:**
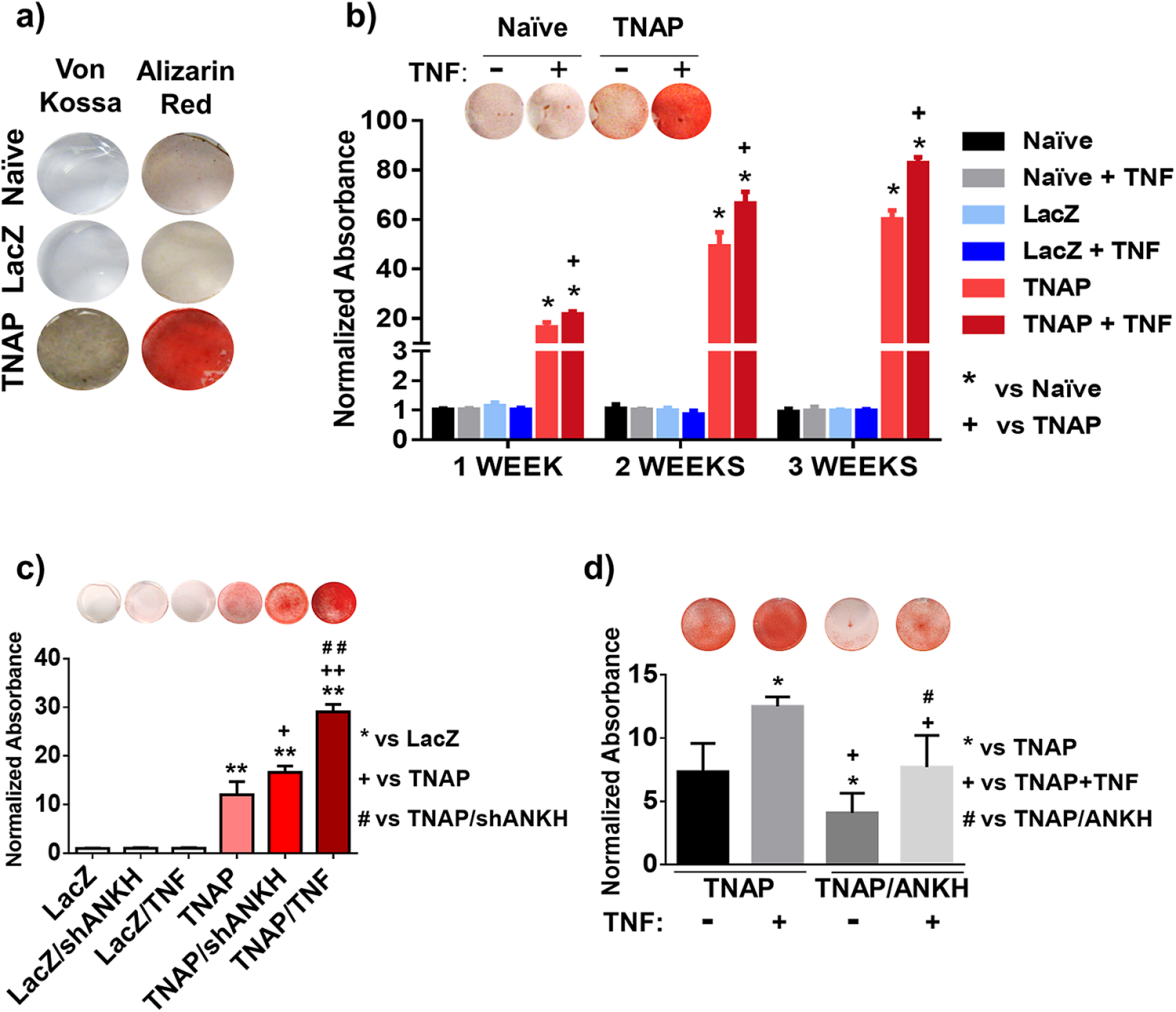
TNF increases mineral deposition in bNP cells. (**a**) Representative image of Alizarin red and von Kossa stained cells: no staining in naïve or LacZ transduced cells. Positive staining in TNAP transduced cells after 3 weeks of incubation in mineralizing medium (n = 3); (**b**) representative image of Alizarin red staining (1 week) and spectrophotometric quantifications of the staining. Increase of mineral deposition observed in cultures of TNAP-transduced bNP cells treated with 20 ng/mL of TNF (n = 4); (**c**) representative image of Alizarin red staining and spectrophotometric quantifications of the staining (1 week). Increase of mineral deposition observed in TNAP/shANKH and TNAP/TNF double transduced bNP cells compared to TNAP single transduced cells (n = 3–5); (**d**) representative image of Alizarin red staining and spectrophotometric quantifications of the staining (1 week). A decrease in mineralization and reduction of TNF-induced mineral deposition when the ANKH gene is over-expressed in TNAP cells (n = 3–5). Significance represents *p < 0.05 or **p < 0.005. Data analyzed using one-way ANOVA.

We first assessed the relative expression levels of TNAP, ANKH and ENPP1 mRNA in primary hNP cells isolated from adult patients (n = 4) pediatric patients (n = 2), and in primary bNP cells (n = 3) via RTqPCR. The results in both hNP and bNP cultures were remarkably similar, with robust endogenous expression of ANKH and ENPP1, and low to undetectable TNAP levels (Fig. 1a). hNP cells were linearly regressed for age vs gene expression, revealing ANKH was significantly more expressed in adult patients (Pearson’s R = 0.9289, p = 0.0074), but ENPP (R = 0.553, p = 0.2551) and TNAP (R = 0.01178, p = 0.9823) had no expression-age effect. Prior investigation into ANKH age-related expression has also shown higher expression in adult prostate tissue compared with adolescents^49^. We then treated hNP cells with exogenous human TNF for 72 h and assessed ANKH and ENPP1 gene expression by RTqPCR. As shown in Fig. 1b, TNF treatment down-regulated ANKH in both adult and pediatric hNP cells, whereas it only reduced ENPP1 expression in pediatric hNP cells (Fig. 1c). Next, to determine whether naïve NP cells have the ability to mineralize their matrix in vitro, we performed Alizarin red and von Kossa stains in bNP cells incubated in mineralization medium for 3 weeks. Naïve bNP cells were negative for Alizarin red and von Kossa stains (Fig. 2a), highlighting the insignificant mineralization potential of these cells, in agreement with their lack of TNAP expression. To assess whether enforced expression of TNAP was sufficient to enable mineralization, we transduced bNP cells with viral expression vectors containing bovine TNAP cDNA (TNAP-bNP). As shown in Supplementary Fig. 1a, TNAP-bNP cells showed robust TNAP expression, assessed by RTqPCR, and strong alkaline phosphatase activity, determined by X-phos staining. Enforced TNAP expression lead to a robust matrix mineralization in TNAP-bNP cells cultured in mineralizing media over a 3-week period (Fig. 2a). Importantly, matrix mineral deposition was further enhanced by TNF in the TNAP-bNP cells, whereas no changes in mineralization were observed in bNP naïve cells treated with TNF (Fig. 2b). These results show that TNAP expression is required for NP mineralization in vitro, and that TNF can further enhance mineralization by modulating the levels or activities of other factors, but TNF treatment of NP cells alone is not sufficient to drive mineralization.

Next, we used viral constructs to stably drive enforced expression of TNF (TNF-bNP cells) or silence ANKH mRNA expression (shANKH) in primary bNP cells (Supplementary Fig. 1b,c). Similar to naïve bNP cells treated with TNF, bNP cells transduced with TNF or shANKH vectors did not show mineralization compared to naïve bNP or LacZ vector controls (Fig. 2c). However, cells double-transduced with TNAP and shANKH vectors (TNAP/shANKH-bNP cells) showed a significant increase in mineralization, compared to TNAP-bNP cells. Moreover, over-expression of TNF in TNAP-bNP cells (TNAP/TNF-bNP) further enhanced the level of Alizarin Red stain detected in TNAP-bNP and TNAP/shANKH-bNP cells (Fig. 2c). Accordingly, stable, enforced expression of ANKH in TNAP-bNP cells (TNAP/ANKH-bNP) (Supplementary Fig. 1e) led to a decrease in mineralization and diminished the effect of TNF stimulation (Fig. 2d). To verify that the composition of the mineral being deposited and stained with Alizarin red in vitro resembled the characteristics of bone tissue and hydroxyapatite (HA), we examined the in vitro mineralized matrix by Fourier transform infrared spectroscopy (FTIR). The results indicated that all three TNAP-transduced cell types (TNAP, TNAP/shANKH, TNAP/TNF) deposited mineral that contained clear phosphate peaks around 1200–900 cm^−1^, carbonyl peaks at 1500 cm^−1^, and amide peaks at 1650 cm^−1^ as found in bone tissue and HA (Supplementary Fig. 2)^50,51^.

### Enforced TNF expression downregulates the expression of inhibitors of mineralization and increases the expression of markers of chondrocyte hypertrophy in primary bNP cells

**Figure 3:**
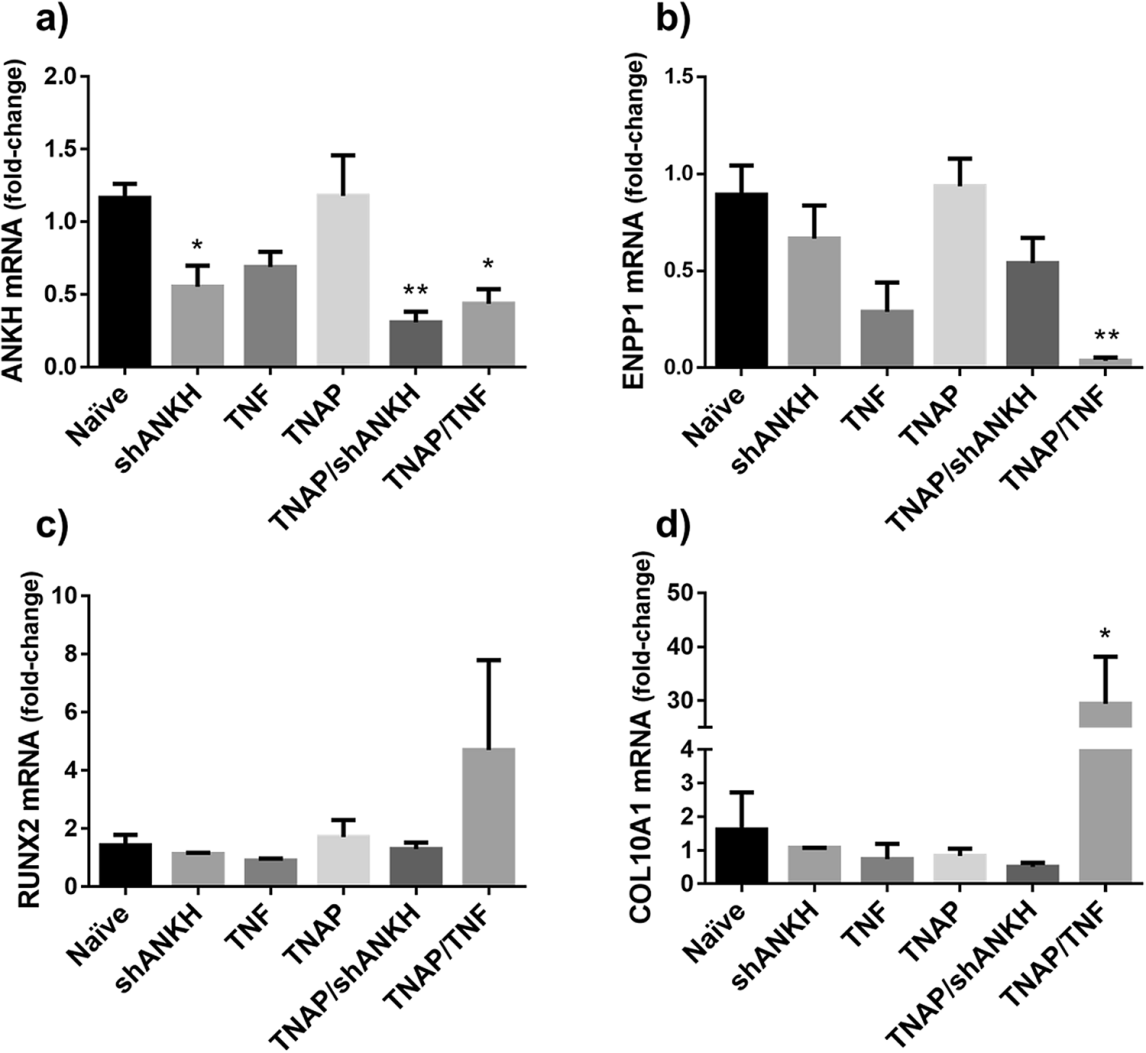
TNF down-regulates inhibitors of mineralization (**a**) ANKH, (**b**) ENPP1 and increases expression of hypertrophy markers, (**c**) RUNX2, (**d**) COL10A1 in TNAP transduced bNPs (n = 3). Data are mean ± S.E.M. of 3 experiments. Significance represents *p < 0.05 or **p < 0.005 compared to naïve cells. Data analyzed using One-way ANOVA.

To evaluate changes in gene expression in the transduced bNP cells, we performed RTqPCR analyses in total RNA isolated from cells cultured for 72 h in mineralization medium. As expected, the shANKH RNA interference construct down-regulated gene expression of ANKH mRNA in both shANKH-bNP and TNAP/shANKH-bNP cells (Fig. 3a). A similar downregulation of ANKH expression was observed in TNF-bNP or TNAP/TNF-bNP cells (Fig. 3a). The expression of ENPP1 was also significantly down-regulated in TNAP/TNF-bNP cells but not in shANKH-bNP or TNAP/shANKH-bNP (Fig. 3b). Expression of type I and II collagen (COL1A2 and COL2A1) was reduced in TNAP/shANKH and TNAP/TNF cells, whereas aggrecan (ACAN) expression remained unchanged. (Supplementary Fig. 3a–c). Markers of chondrocyte hypertrophy, RUNX2 and COL10A1, were up-regulated only in TNAP/TNF cells (Fig. 3c,d), whereas MMP13 expression was not significantly modulated, and showed a trend towards reduced expression in all TNAP-transduced groups (Supplementary Fig. 3d). The observed effects were not due to the transduction process as no significant changes in gene expression were detected in LacZ- or shGFP-transduced cells (Supplementary Fig. 4). Confirmatory protein expression level of collagen type X was shown to reflect mRNA expression changes (392 ± 19 Naïve vs. 771 ± 168 TNAP/TNF, p < 0.0001), as assessed by immunofluorescence histology (Supplementary Fig. 5). Together, these results show that the hypermineralizing TNAP/TNF-bNPs display a chondrocyte hypertrophy-like phenotype, concomitant with decreased expression of inhibitors of mineralization.

### Inhibition of the NF-κB pathway diminishes TNF-enhanced mineralization

**Figure 4:**
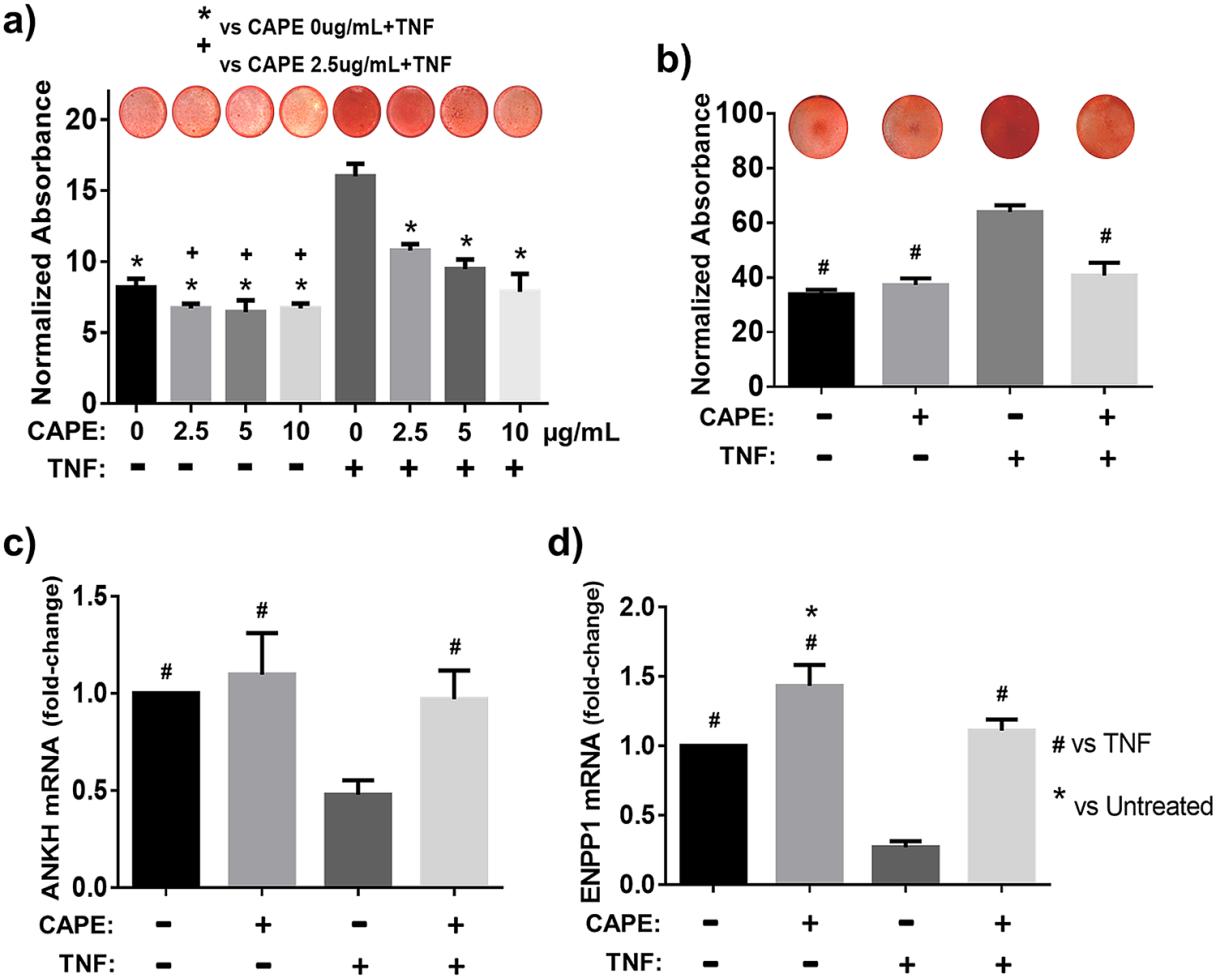
Inhibition of NF-κB pathway diminishes TNF-induced mineralization. (**a**) Representative image of Alizarin red staining and spectrophotometric quantifications of the staining (1 week): pretreatment of TNAP cells for 1 h with CAPE diminished the TNF effect on mineral deposition in a dose-dependent matter (n = 4); (**b**) representative image of Alizarin red staining and spectrophotometric quantifications of the staining (2 weeks) (n = 6); (**c**) qPCR results: pretreatment of TNAP cells for 1 h with 5 µg/mL CAPE diminished the effect of TNF on ANKH and (**d**) ENPP1 gene expression (n = 4). Significance represents p < 0.05. Data analyzed using One-way ANOVA.

The nuclear factor kappa B (NF-κB) pathway modulates TNF-induced actions in different contexts^52,53^, including decreasing ANKH in hASMC via p65 canonical signaling^21^. Thus, to investigate the involvement of NF-κB signaling in the TNF-driven mineralization of transduced bNP cells, in vitro cultures were pretreated with caffeic acid phenethyl ester (CAPE), a specific and potent inhibitor of the activation of canonical p65 NF-κB signaling^54,55^. Pretreatment of TNAP-bNP cells with CAPE diminished the TNF-induced enhancement of mineral deposition in a dose-dependent manner in 1-week cultures (Fig. 4a), and CAPE pretreatment completely abolished mineralization in 2-week cultures treated with TNF, which showed Alizarin red stain levels comparable to untreated TNAP-bNP controls (Fig. 4b). Using RTqPCR, we examined the effect of inhibiting NF-κB signaling on the ANKH and ENPP1 gene expression. As shown in Fig. 4c,d, CAPE pretreatment blocked the TNF-induced decrease in ANKH and ENPP1 expression and led to increased ENPP1 basal expression in vehicle-treated bNPs.

### Increased mineralization in discs injected with bNP-transduced cells

**Figure 5:**
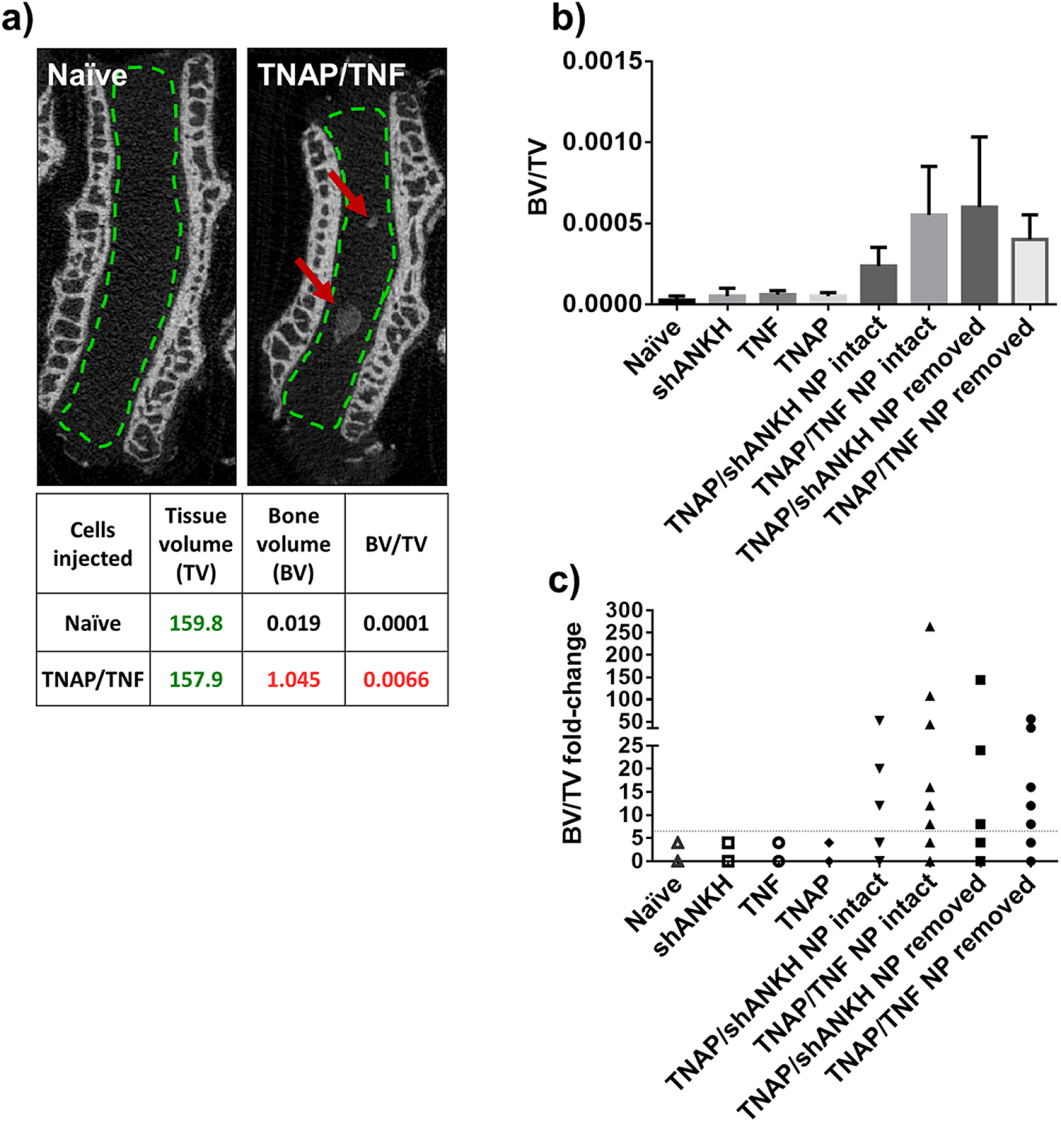
Mineral content increased in IVD injected with TNAP/TNF transduced bNPs. (**a**) µCT image of rabbit discs injected with naïve and TNAP/TNF double-transduced cells; The tissue volume (TV), bone volume (BV) and new mineral formation (BV/TV) for the representative images are given in the table below the image; (**b**) new mineral formation in IVD injected with transduced cells assessed by µCT; the results presented as mean and standard error of all discs in a given group; (**c**) BV/TV of discs injected with transduced cells represented as a fold-change, compared to mean BV/TV in disc injected with naïve bNPs (n = 8/group).

To assess whether the changes in phenotype and ability to mineralize of the modified bNPs in vitro was functionally relevant, we used an ex vivo organ culture system. To this end, we injected genetically manipulated bNP cells into rabbit disc cultures to examine their ability to mineralize in a native tissue environment. We assessed mineralization at 28 days post-injection by micro-computed tomography (μCT) (Fig. 5a). We observed high variation in the amount of newly formed mineral in the injected groups, assessed by measuring the bone volume fraction (bone volume/tissue volume—BV/TV); however, mineral formation was observed only in discs that were injected with TNAP/TNF and TNAP/shANKH cells (Fig. 5b). We did not detect changes in mineralization in discs injected with naïve, TNF-, shANKH-, or TNAP-bNPs. Further, μCT results revealed that the group injected with TNAP/TNF cells had the highest percentage (35%) of discs with increased BV/TV, whereas 27% of discs injected with TNAP/shANKH had a detectable increase of BV/TV. In discs where the NP was aspirated prior to injection, we observed a much higher incidence of BV/TV increase (67% of the discs injected with TNAP/TNF cells and 50% of discs injected with TNAP/shANKH) (Fig. 5c; Supplementary Fig. 6) indicating that it was advantageous to remove the native disc material to promote mineralization.

## Discussion

The healthy NP is a tissue non-permissive to mineralization. While spontaneous mineralization of NP tissue is a pathological event associated with IDD^1–4^, inducing mineralization and bone production within the IVD where the NP would reside is a desired outcome of anterior spinal fusion surgery. In this study we aimed to better understand the mechanisms sufficient to confer NP cells the ability to mineralize their matrix, with the ultimate intention to develop systems with potential clinical therapeutic application. Specifically, we studied the role that TNF plays in regulating the expression of ANKH and ENPP1, two inhibitors of mineralization highly expressed by NP cells, and how TNF works to promote NP matrix mineralization in vitro and ex vivo. We showed that TNF inhibits the expression of ANKH and ENPP1 by NP cells, and enhances the matrix mineralization by bNP cells stably transduced with TNAP-expressing viral vectors. We demonstrated that the TNF-dependent mineralization and ANKH/ENPP mRNA suppression were blocked by the NF-κB p65 inhibitor CAPE. Importantly, we also showed in an ex vivo organ culture model that injection of modified bNP cells into the NP of an intact IVD promotes mineralization of the normally mineralization resistant NP. Taken together, our results provide insight into the mechanisms sufficient to drive NP matrix mineralization within an intact IVD, and represent a step towards a potential therapeutic intervention: promotion of non-surgical IVD fusion using a percutaneous injection into the IVD space.

We found that human and bovine primary NP cells have high levels of inhibitors (ANKH and ENPP1) and low or undetectable levels of facilitators (TNAP) of matrix mineralization, which agrees with the natural resistance of the NP to mineralize, and findings from many other non-mineralized tissues^11^. Interestingly, we observed higher expression of endogenous ANKH in hNP cells from adult patients compared to cells obtained from pediatric patients, in agreement with previously published results in prostate specimens^49^, a finding that seems to contradict higher rates of mineralization in older IDD patients^3,4^. However, it must be understood that gene expression in non-mineralizing-IDD vs. mineralizing-IDD discs may be different, such as increased TNAP^1,56^ and decreased ANKH/ENPP^56^ in the IVD of mineralizing discs, and that these gene expression changes correlated with advanced disc degradation and pathologic mineralization events. We overexpressed TNAP in bNP cells using retroviral vectors emulating the increased TNAP levels observed in IDD. Overexpression of TNAP was enough to induce matrix mineralization in tissue culture, suggesting that TNAP activity alone is sufficient for NP cells to mineralize in vitro. However, these TNAP-overexpressing cells failed to mineralize IVDs ex vivo, suggesting that additional factors, such as high expression of ANKH or ENPP1, prevented mineral deposition. We also observed that either TNF treatment or enforced TNF expression further enhanced the mineral deposition in TNAP-overexpressing bNPs in vitro. Our gene expression analysis showed that TNF decreased ANKH mRNA by approximately 50%, in agreement with reports showing that TNF promotes ectopic mineralization in hASMCs by suppressing ANKH mRNA expression by half^21^, and other authors in several tissues showing induced matrix mineralization and 50% reduction of ANKH mRNA expression in target cells as a result of TGF-β1^57^, ePi/forskolin^58^, or IL-1β^24,25^ treatment, whereas basic fibroblast growth factor (bFGF)^59^ and IL-6^60^ treatment increased ANKH mRNA by twofold while still driving mineralization. Regarding ENPP1 expression modification by cytokines, it has been reported that IL-1β decreases ENPP in hMSCs^23^ and in bNPs^25^, mRNA for ENPP was also reduced in rat ASMCs by ePi/forskolin treatment^58^, and ENPP is decreased in IDD patients with advanced degeneration and mineralization^56^. Treatment of human deciduous teeth stem cells with IL-6 had little effect on ENPP1 mRNA expression, whereas bFGF resulted in nearly a doubling of baseline levels^59,60^, and rat endplate chondrocytes increase ENPP mRNA following TGF-β1 treatment^61^. There appears to be a complicated tissue- and factor-specific influence on ANKH and ENPP expression modulation.

In keeping with these observations, bNPs stably expressing shANKH and TNAP showed increased mineralization ability compared to naïve or TNAP-expressing cells. However, the increased mineralization potential of TNAP/shANKH-bNP was lower than that of TNAP/TNF-bNPs. The latter suggested that TNF modulation of factors other than just ANKH was required to further enhance mineralization in NP cells, which was confirmed by the decrease of both ANKH and ENPP1 expression observed in the hypermineralizing TNAP/TNF-bNPs. Accordingly, overexpression of ANKH reduced mineral deposition in TNAP-overexpressing bNPs and also blocked the enhanced mineralization induced by TNF in these cells, where TNF would only be able to efficiently down regulate ENPP1. Importantly, ex vivo injection of IVDs with TNAP/TNF- or TNAP/shANKH-bNPs lead to mineral formation in the NP, with much higher mineral formation observed in TNAP/TNF injected groups. This finding further supports the idea that the inflammatory milieu can drive disc mineralization by decreasing both ANKH and ENPP1 expression. Together, these results indicate that high levels of one mineralization inhibitor is sufficient to decrease the mineralization potential of NPs, which agrees with studies by Harmey et al.^13^ using ENPP1/TNAP double-deficient mice and by Hessle et al.^14^ using ANKH/TNAP double deficient mice, where each study showed that the more severe ectopic mineralization seen in each of the ENPP or ANKH single knockout mice is more normalized in the double knockout animals. It is important to point out the high variability in mineral deposition we observed in the injected discs ex vivo, and that mineralization was significantly enhanced by the partial removal of a portion of the native NP matrix, similar to prior reports utilizing a pig model with partial disc removal^44^. We observed that the IVD mineral formed was punctate and did not span the whole disc space 4 weeks after injection, suggesting that mineralization occurs only in the immediate surrounding of the injected cells. The latter could be due to the anti-osteogenic properties of the native NP matrix which, in addition to expressing ANKH and ENPP1, would also contain other inhibitors of mineralization such as Matrix Gla protein and Osteopontin^62–64^. It should also be noted that although the TNF expression used in our model would be expected to diffuse and potentially alter ANKH/ENPP expression in native IVD cells making them more prone to allow mineralization, the TNAP being overexpressed is matrix bound, and its effect would be expected to be spatially constrained. Therefore, the contribution of other inhibitors on NP matrix mineralization should also be studied, as well soluble forms of TNAP or alternate methods to induce endogenous TNAP expression in the resident IVD NP cells, in order to further improve our ability to enhance NP mineralization and potentially IVD fusion.

Our results using CAPE, a pharmacological inhibitor of p65 NF-κB signaling^55^, showed that the TNF-driven ANKH and ENPP1 downregulation in bNPs is NF-κB-dependent, and is in agreement with prior reports in hASMCs^21^. TNF-induced NF-κB signaling appears to have a large role on the inflammation-driven IVD degeneration^18,20,65–69^. NP cells from patients with IDD show increased NF-κB activity, along with increased levels of inflammatory mediators (IL-6) and matrix-degrading enzymes (MMP-3, 9, and 13, and ADAMTS-4 and 5), and reduced aggrecan and collagen type II expression^67^. Importantly, these studies showed that the degree of disc degeneration was directly correlated with the level NF-κB activation^66,67^. In addition to NF-κB, results in human specimens and animal models suggest that RUNX2 induction is a central contributing factor to the pathogenesis of IDD, patients with IDD showed increased RUNX2 and COL10A1 mRNA and protein levels in their discs^1,70,71^, and increased RUNX2 and collagen type X protein levels were only observed in IDD cases of severe degeneration, correlating with the highest levels of calcification^1^. Similarly, Runx2 mRNA levels were significantly upregulated in mouse models of IVD degeneration^70^. In addition, studies using discs from IDD patients or TNF-treated bovine discs showed decreased ACAN, COL2A1 and COL1A2 mRNA expression^19,20,72^. Our results mirror these observations in human and animal models of IDD. In addition to having decreased expression of ANKH and ENPP1, we found that TNAP/TNF-bNPs showed decreased ACAN, COL2A1 and COL1A2 mRNA, and increased expression of RUNX2 and COL10A1. Thus, our data further supports the notion that RUNX2 could contribute to IDD pathogenesis, perhaps mediating the actions of inflammatory cytokines to drive phenotypic changes in the NP cells. The resulting upregulation of COL10A1 could therefore enable mineralization in the normally non-mineralizing disc by associating to matrix vesicles to which TNAP is membrane bound^73^.

There is a tremendous amount of development left to complete, but if the current method of delivering NP cells overexpressing TNAP/TNF were to be applied clinically now, there would appear to be a clear risk for inducing rapid IDD and discogenic pain, not to mention local escape of induced bone from the IVD receiving treatment, that could lead to spinal stenosis or other entrapment pathologies. TNF induces several genes contributing to IDD as already mentioned, and it has also been shown to increase expression of nerve growth factor (NGF)^74,75^ and IL-6^76^. NGF is a neurotrophin that supports nerve growth and substance p expression in vitro^77,78^, and may contribute to painful sensory nerves penetrating and growing into the disc. IL-6 is a cytokine known to drive inflammatory processes, but specific to NP cells it increases expression of IL-1β and TNF causing amplification their effects^79^, and contributes to increasing IVD expression of at least vascular endothelial growth factor (VEGF) and brain derived neurotrophic factor (BDNF)^80^. BDNF would strengthen the neurite ingrowth into the IVD described above for NGF, and VEGF would be expected to both promote and support angiogenesis into the IVD^80^, but perhaps more problematic, VEGF would also act as a survival and trophic factor for NP cells^81^, potentially making the native NP cells less likely to respond to the pro-osteogenesis treatment being delivered. All of these untoward effects are secondary to the TNF, and could be eliminated if the gene-programmed NP cells could be made to be identically or more mineralizing/osteogenic than the TNF-containing treatment. The early data presented here shows that shANKH/TNAP is almost as vigorous at promoting NP matrix mineralization as TNF/TNAP, and it could be suggested that a combined shANKH/shENPP/TNAP may be equal to or more mineralizing than TNF/TNAP. The issue of bone formation external to the disc space is not a TNF-dependent issue, and would potentially be a problem in the above suggestion for double RNA-interference too. It seems that there needs to be means to stop the treatment cells from mineralizing their local matrix as soon as they exit the IVD space. We suggest using allogeneic or xenogeneic cells for implantation, and then in an immunocompetent host the grafted cells would meet an immediate immune response that would kill them and clear their pro-mineralization effects. The central portion of the IVD is known to be devoid of vascularity, and is therefore not subject to the typical immune system’s surveillance. The grafted cells would be able to work for a period of time making the interior of the disc mineralized and bone-like, the degree of which would be affected by how vigorous and effective the implanted cells were capable of being, prior to being detected. Prior research with intramedullary stabilizing xenografts vs. stainless steel K-wires of experimentally fractured humeri in birds^82^, using xenografts to heal rabbit radial bone defects^83^, and xenografts used clinically^84^ have all shown that xenografts are capable of achieving bone growth and healing, despite inflammation that forms due to host/graft responses. The degree to which xenograft-derived inflammation drives pain at the treatment injection site or possibly interferes with progression towards the fusion desired will need to be assessed in the future when those assessments are performed.

We recognize that there are limitations to the current work. Our findings regarding bovine and human gene expression levels are based on a limited number of samples, particularly the age-dependence expression of ANKH, and although our findings agree with the reports of other investigators and different tissues, examination of a larger number of samples would have strengthened our study. Our gene-regulation data is based upon mRNA perturbations and inhibitor-dependent effects, and other than for the collagen type X immunostaining, was not corroborated with protein expression data to prove that the mRNA-level regulations reflect similar protein-level effects. We speculate that the shENPP down regulation of ENPP1 would mimic the effects of shANKH that are reported here, and that their combination (shANKH/shENPP) would better mimic TNF/TNAP as regards upregulation of RUNX2 and COL10A1 not seen in shANKH, and possibly better mimic the NP phenotype shift towards hypertrophic chondrocyte that was observed. Lastly, our ex vivo results in intact IVD disc organs supporting the establishment of intra-discal mineralization were not exposed to the rigors of an intact animal, including possible immunological response or other physiological homeostatic mechanisms that are not represented in the disc organ culture setting. These limitations are planned to be addressed in future work in developing the model.

Our study provides novel data addressing the effect of proinflammatory cytokines on NP cells, as regards gene-programming the cells sufficiently to make them capable of vigorously mineralizing their matrix. Both bovine and human primary NP cells showed similar changes in ANKH and ENPP1 expression upon treatment with TNF in vitro, which in turn led to an increase in bNP matrix mineralization. This effect was mediated by the activation of the p65 NF-κB signaling pathway, which could at least in part explain the pathologic calcification of IVDs observed during degeneration. By leveraging the mechanism/s by which pathologic mineralization occurs in the disc, it may be possible to develop a minimally invasive, non-surgical therapeutic approach for patients requiring spinal fusion. This model could be very useful for developing strategies to induce fusion in the soft-tissue disc space and provides great insight into the regulation of the chemical moieties responsible for mineral formation. Future studies using a mouse model of IDD can be performed to provide further mechanistic insight and to better understand the contribution of the inflammatory milieu in the progression of disc mineralization.

## Methods

### Primary bovine and human NP cell isolation

Bovine NP (bNP) cells were obtained from three cadaveric bovine tails of young adult animals (18–36 months old) (Cohen Max Insel Animal Organs and Tissues for Research, Livingston, NJ). The tails were obtained, and experiments performed, following approval of the Hospital for Special Surgery (HSS) Institutional Animal Care and Use Committee (IACUC) and in compliance with ARRIVE guidelines, with the protocol considered exempt because the tails were commercially obtained from cadaveric specimens euthanized off site. All samples were handled in accordance with HSS Comparative Lab Animals Services (CLAS) guidelines and regulations. bNP cells were isolated as previously described^85^. The discs were dissected, the endplates were removed, and NP tissue was extracted using an 8 mm biopsy punch. Primary bNP cells were isolated by initially digestion in 0.19% pronase (Roche) solution for 1 h with a subsequent overnight digestion in 500 U/mL Collagenase Type II (Worthington Biochemical Corporation, Lakewood NJ) at 37 °C. After digestion, cells were washed and seeded at 2.8 × 10^4^ cells/cm^2^ density in complete medium, consisting on high glucose Dulbecco’s Modified Eagle Media (DMEM; Gibco, Grand Island, NY), 10% Fetal Bovine Serum (FBS; Gibco), 1% antibiotic–antimycotic (Gibco) and 10 µM HEPES buffer (Gibco), and incubated in a humidified atmosphere of 5% CO_2_. Cells were used for experiments at passage 2.

Human NP (hNP) cells were isolated from six patients (Table 1) undergoing elective surgical procedure under an HSS Institutional Review Board (IRB) approved study (HSS IRB protocol #12037). All experiments were performed in accordance with all institutional guidelines and regulations, and informed consent for sample collection was acquired from each patient and/or their legal guardian. Preoperative imaging did not demonstrate mineralization on plain X-rays for any of the patient donors. Patients were grouped as pediatric (ages up to 18 years old), or adult (ages 18 years and older). hNP tissue was explanted and categorized (NP, AF, cartilage endplate, or other) by the surgeon, and then transported on ice to the research lab for processing. Samples from multiple levels were pooled into a single culture. hNP tissue was digested with 200 U/mL of collagenase type II following the protocol previously described for bNP cell isolation. Passage 1 hNP cells were cultured until 90% confluence, and then cells were collected for total RNA isolation and RT-qPCR analyses of endogenous ANKH, ENPP1 and TNAP expression. Passage 2 hNP cells were seeded at 130 × 10^3^ cells/cm^2^ in Falcon 12-well plates (BD Biosciences). Upon reaching 70% confluency, cells were serum-deprived for 24 h before treatment with 20 ng/mL of TNF (R&D Systems, Minneapolis, MN), or vehicle. At 72 h after treatment with TNF, total RNA was extracted for RTqPCR analyses (n = 4).

**Table 1:** List of human donors.

Human ID #	Diagnosis	Revision	Gender	Age at explant	Disc level	Pfirrman	Harvest
1	DDD, flatback	Y	M	57	L2/3	III	Anterior
					L3/4	II	Anterior
2	DDD, flatback	Y	F	55	L2/3	II	Anterior
					L3/4	IV	Anterior
					L4/5	III	Anterior
3	HNP	N	M	70	L5/S1	N/A	Posterior
5	DDD, scoliosis	N	F	59	L1/2	V	Anterior
					L2/3	IV	Anterior
7	Spondylolisthesis	N	F	12	L5/S1	IV	Posterior
8	Spondylolisthesis	N	F	15	L5/S1	III	Posterior

### Retroviral constructs

Passage 2 bNP cells were stably transduced using in-house generated pMXs-IRES-Bsd-TNAP, pMXs-IRES-Neo-TNF, pMXs-IRES-Neo-ANKH or pMXs-U6-Puro-shANKH retroviral constructs. pMXs-IRES-Bsd-LacZ and pMXs-U6-Puro-shGFP constructs were used to assess transduction efficacy. The retroviral transductions were performed by spinoculation with amphotyped viruses prepared with Phoenix A packaging cells as described before^86^. Briefly, in the presence of 8 µg/mL polybrene viral supernatants were applied to 70% confluent cells by centrifugation at ~ 1100*g* at 32 °C for 45 min followed by 5 h incubation at 32 °C in 5% CO_2_. The cells were then switched into complete medium and depending upon the retroviral vector utilized, transduced cells were selected using 4 µg/mL Blasticidin (Bsd constructs), 400 µg/mL G418 (Neo constructs), or 1 µg/mL Puromycin (Puro constructs), alone or in combination, in single or double-transduced bNPs respectively. pMXs-IRES-Bsd, pMX-IRES-Neo, pMXs-U6-Puro were purchased from Cell Biolabs, Inc, San Diego, CA, whereas TNAP, TNF, ANKH genes were obtained from OriGene Technologies, Inc., Rockville, MD.

### Cell culture

Naïve and transduced bNP cells were seeded at 130 × 10^3^ cells/cm^2^ in Falcon 12-well plates (BD Biosciences). Cells were maintained in mineralization medium, which consisted of complete medium supplemented with 5 mM β-glycerophosphate (Sigma-Aldrich, St. Louis, MO) and 50 µg/mL l-Ascorbic acid (Sigma-Aldrich), in the presence or absence of 20 ng/mL of TNF (R&D Systems, Minneapolis, MN), for 1, 2, and 3 weeks, with medium changed every 3 days.

For experiments involving NF-κB inhibition, cells were pretreated for 1 h with 0, 2.5, 5 or 10 µg/mL of Caffeic Acid Phenethyl Ester (CAPE; Calbiochem), a potent inhibitor of NF-κB pathway activation^54^, and then treated with 20 ng/mL of TNF and cultured for 1 or 2 weeks of incubation in mineralization medium.

### In vitro staining

Alizarin red staining was performed and quantified as described by Gregory et al.^87^. Briefly, cells were fixed in 10% neutral buffered formalin (Sigma-Aldrich) for 10 min, rinsed three times (5 min each) in distilled de-ionized (dDI) water, and stained with 2% alizarin red solution (Sigma-Aldrich) at pH 4.1- 4.3 for 20 min. The plates were then rinsed three times (10 min each rinse) in dDI water and imaged by a full plate scan with a color scanner. After imaging, the alizarin red dye bound to calcium was quantified on a TECAN SpectraFluor Plus photospectrometer (Mannedorf, Switzerland) at 405 nm, after 10% acetic acid dissolution and 10% ammonium hydroxide quenching.

Von Kossa staining was performed by fixing cells as described above and incubating them with 5% silver nitrate solution (Sigma-Aldrich) for 40 min; during staining cells were exposed to 100-W white light. Plates were then rinsed twice with dDI water, incubated with 5% sodium thiosulfate for 1 min, and imaged with a color scanner.

X-Phos staining was performed according to the manufacturer’s protocol by fixing cells as described above and incubating them for 1 h at 37 °C with X-Phos 1-step NBT/BCIP Solution (Thermo Scientific).

### Pellet cultures and RT-qPCR analysis

3-Dimensional (pellet) bNP cultures were used to assess gene expression^86^. After trypsinization, 10^6^ cells were spun down 3 times at 4 °C at 2000 rpm, with medium changed every time. The cells were then incubated in 1.6 mL tubes for 72 h at 37 °C in a humidified atmosphere of 5% CO_2_ in complete medium. After the pellets were formed, the medium was changed to mineralization medium and pellets were incubated for additional 72 h before RNA isolation.

### RNA isolation and RT-qPCR analyses

Total RNA was extracted using TRIzol reagent (Life Technologies) followed by DNaseI treatment and column clean-up (QIAGEN). RNA was reverse transcribed using the iScript Reverse Transcription Kit (Biorad). Amplifications were carried out using SYBR Green I-based RT-PCR on the Opticon 2 Real Time PCR Detector System (BioRad), using PCR primers specific for TNAP, TNF, ANKH, ENPP1, RUNX2, MMP13, COL10a1, COL2a1, COL1a2 and ACAN (Table 2). Cycling parameters were: initial denaturation 95 °C for 3 min, then 39 cycles of: denaturation at 95 °C for 10 s, annealing at 5 °C lower than lowest primer used for 10 s, and extension at 72 °C for 30 s. Data were calculated as the ratio of each gene to RPL13a, using the 2^−ΔΔCt^ method for relative quantification^88^.

**Table 2:** List of oligonucleotides.

Gene	Gene ID	Amplicon size (bp)	Seq. forward (5′ > 3′)	Fwd Tm (°C)	Sequence reverse (5′ > 3′)	Rev Tm (°C)
Bovine ANKH	511800	128	CCA TGT GGA TGA GTC AGT GG	55	GCA CAT CCA ACC AGG AAA CT	55.4
Bovine ENPP1	615535	159	AAT TGA GCG CTT GAC GTT CT	55.6	TCA GTG CTG TGC TTG AAT CC	55.6
Bovine COL1A2	282188	69	ACA TGC CGA GAC TTG AGA CTC A	58.1	GCA TCC ATA GTA CAT CCT TGG TTA GG	57.3
Bovine COL2A1	497142	125	GCT TCC ACT TCA GCT ATG GA	54.4	CAG GTA GGC AAT GCT GTT CT	54.9
Bovine TNAP	280994	291	GCC GGG GGA CAT GCA GTA CG	54.9	GCC GGG GGA CAT GCA GTA CG	54.8
Bovine TNF	280943	165	AGA GGG AAG AGT TCC CCA GG	57.2	CCT CAG CTT GAG GGT TTG CT	57.3
Bovine ACAN	280985	150	GGG AGG AGA CGA CTG CAA TC	57.3	CCC ATT CCG TCT TGT TTT CTG	54.4
Bovine RUNX2	536911	62	AGT GAT TTA GGG CGC ATT CCT	56.6	GAG GGC CGT GGG TTC TG	57.3
Bovine COL10A1	282416	225	GGA AAA CAA GGG GAG AGA GG	54.5	TCC CCT TTC TGT CCA TTC AG	53.8
Bovine MMP13	281	104	TCC AGT TTG CAG AGA GCT ACC	56.6	CTG CCA GTC ACC TCT AAG CC	57.2
Human ANKH	914	128	CCA TGT GGA TGA GTC AGT GG	55	GCA CAT CCA ACC AGG AAA CT	55.4
Human ENPP1	5167	150	AAT TGA GCG CTT GAC GTT CT	55.6	TCA GTG CTG TGC TTG AAT CC	55.6
Human TNAP	249	275	GGA CAT GCA GTA CGA GCT GA	56.9	CCA CCA AAT GTG AAG ACG TG	45.5

### Ex vivo disc organ culture

IVDs were isolated from adult New Zealand White rabbits as described previously^89^. All animals were obtained, and experimental protocols were performed, following approvals by the HSS IACUC and in compliance with ARRIVE guidelines. The cadaveric animal spines utilized in this study were obtained after the animals had been euthanized and discarded by independent investigators performing independent protocols. Cadaveric samples were handled in accordance with HSS CLAS guidelines and regulations. Briefly, lumbar motion segments were dissected, posterior elements, soft tissues and vertebras were removed allowing isolation of IVDs with endplates intact. IVDs were freely suspended in high glucose Dulbecco’s Modified Eagle Media (DMEM; Gibco, Grand Island, NY), 10% Fetal Bovine Serum (FBS; Gibco), 1% antibiotic–antimycotic (Gibco), 10 µM HEPES buffer (Gibco) and 50 µg/mL l-Ascorbic acid (Sigma-Aldrich), and incubated under standard culture conditions (37 °C, 5% CO_2_). The next day the IVDs were injected through the annulus with naïve, LacZ, TNAP, shANKH, TNF, TNAP/shANKH or TNAP/TNF-transduced cells (8 × 10^6^ cells in 30 µL of complete medium/condition) using 25 gauge needle (Becton Dickinson). To decrease the pressure and increase retention of injectate, a portion of the discs underwent partial nucleotomy, by aspirating NP tissue with a 25 gauge needle before cells injection. Discs were incubated at 37 °C for 28 days in mineralization medium, with medium changed every 3 days prior to µCT analysis.

### Micro-computed tomography (μCT)

After fixation in 10% neutral buffered formalin (Sigma-Aldrich) over night at 4 °C, IVDs were transferred to 70% ethanol for short-term storage and analyzed by μCT. For micro-CT analysis, a Scanco μCT 35 (Scanco Medical, Brüttisellen, Switzerland) system was utilized. Imaging was performed with 6 μm voxel size, at 55KVp, 0.36° rotation step (180° angular range) and a 400 ms exposure per view. Scanco μCT software (HP, DECwindows Motif 1.6) was utilized for image analysis, 3D reconstruction, and thresholding. After 3D reconstruction, volumes were segmented using a global threshold of 0.4 g/cm^3^. Bone morphometrics were measured in the NP and AF only (the interior space of the soft IVD with the endplates exclusion as indicated by green, dashed line in Fig. 5a). Bone volume (BV), tissue volume (TV), directly measured bone volume fraction (BV/TV, voxels/mm^3^).

### FTIR analysis

Passage 2 bNP cells were plated at 130 × 10^3^ cells/cm^2^ and cultured for 14 days in mineralization medium. After 14 days, culture medium was aspirated, cells were dried and ground into a fine powder in 200 mg of Potassium bromide (KBr). The KBr/mineral mixture was then compressed to form pellets which were analyzed by FTIR spectroscopy using established techniques^50^. Infrared light from 400–4000/cm^−1^ was passed through the sample and the absorbance was recorded. Mineral composition was assessed by examining magnitude of the area of the amide 1 peak (1710–1590), mineral peak (1215–900), carbonate peak (852–890), HA crystallinity peak (500–670), and the crosslink intensity (1660/1690 ratio), crystallinity intensity (1030/1020 ratio), and acid phosphate (1128/1096 ratio). These were compared to bone and hydroxyapatite control samples.

### Immunofluorescence staining and analysis

Triplicate bNP pellets were cultured for each condition for 72 h in mineralizing media, collected and embedded in Tissue-Tek O.C.T. compound. 8 µm frozen sections were fixed in cold acetone (− 20 °C), blocked with 5% BSA for 30 min at room temperature, and incubated with primary antibody against collagen X (Col10, Abcam) overnight at 4 °C. The sections were then incubated with Alexa Fluor 555 conjugated secondary antibodies (Cell Signaling) for 2 h at room temperature and mounted using ProLong Gold antifade medium with DAPI (Life Technologies). Images were captured using a Nikon Eclipse Ni-E microscope, and the Col10-positive mean pixel density was measured and normalized to the DAPI+ signal in multiple locations of at least 3 sections of each specimen. Signal levels were set to a threshold level based on isotype-matched IgG staining of the sections (data not shown), and are reported as averages ± standard deviation.

### Statistical analysis

All data are expressed as the mean ± SEM (error bars) of at least 3 independent experiments. Statistical analysis was done using GraphPad Prism 6.01 statistical software (GraphPad Prism Software, Inc., La Jolla, CA). Data were compared using one of several different statistical tests including One-way ANOVA or Student’s *t* test, with the choice of statistical test depending on unique details or aspects of each experiment, as indicated in the Figure Legends.

## Supplementary Information

Supplementary Figures.

## Data Availability

Data generated or analyzed during this study are available from the corresponding author on reasonable request.
