# Supplementary material for: Activation of nuclear factor-kappa B by TNF promotes nucleus pulposus mineralization through inhibition of ANKH and ENPP1
Source: Sci Rep. 2021 Apr 15;11:8271. doi: 10.1038/s41598-021-87665-2 (PMC8050288; doi:10.1038/s41598-021-87665-2)
Supplement: Supplementary file 1 — Supplementary Figures. [file 41598_2021_87665_MOESM1_ESM.docx]

**Activation of nuclear factor-kappa B by TNF promotes nucleus pulposus mineralization through inhibition of ANKH and ENPP1**

Agata K. Krzyzanowska MSc^a^, Robert J. Frawley PhD^a, b^ Sheela Damle MD PhD^a^, Tony Chen PhD^a^, Miguel Otero PhD^a^, Matthew E. Cunningham, MD-PhD^a,c*^

^a^Hospital for Special Surgery, New York City, New York, USA
HSS Research Institute, 515 E 71^st^ Street, New York, NY 10021
^b^Weill Cornell Graduate School of Medical Sciences, New York City, New York, USA
1300 York Avenue, LC501, New York, NY 10065

c Weill Cornell Medical College, New York City, New York, USA
1300 York Avenue, LC501, New York, NY 10065


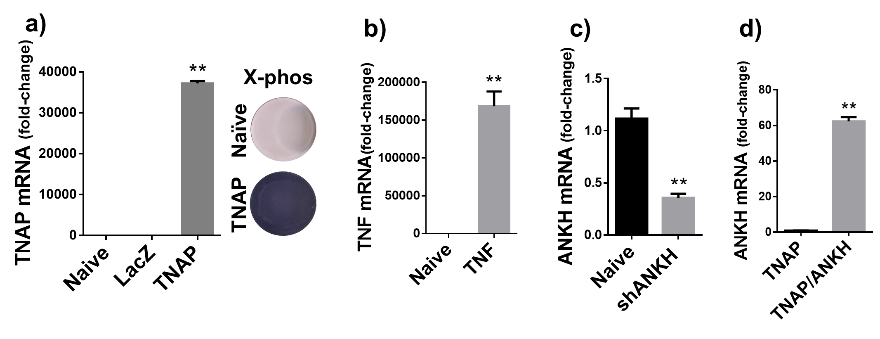


Supplementary Figure 1. Assessment of gene manipulation in bNP cells. a) Increase of TNAP mRNA and Alkaline Phosphatase activity (positive X-phos staining) in cells transduced with TNAP; b) qPCR results confirmed overexpression of TNF, c) knock-down of ANKH and d) overexpression of ANKH in bNPs transduced with retrovirus (n=3). Significance represents p<0.005 (**). Samples in Supplementary Figure 1b), c) and d) were analyzed using unpaired, two-tailed Student t test.


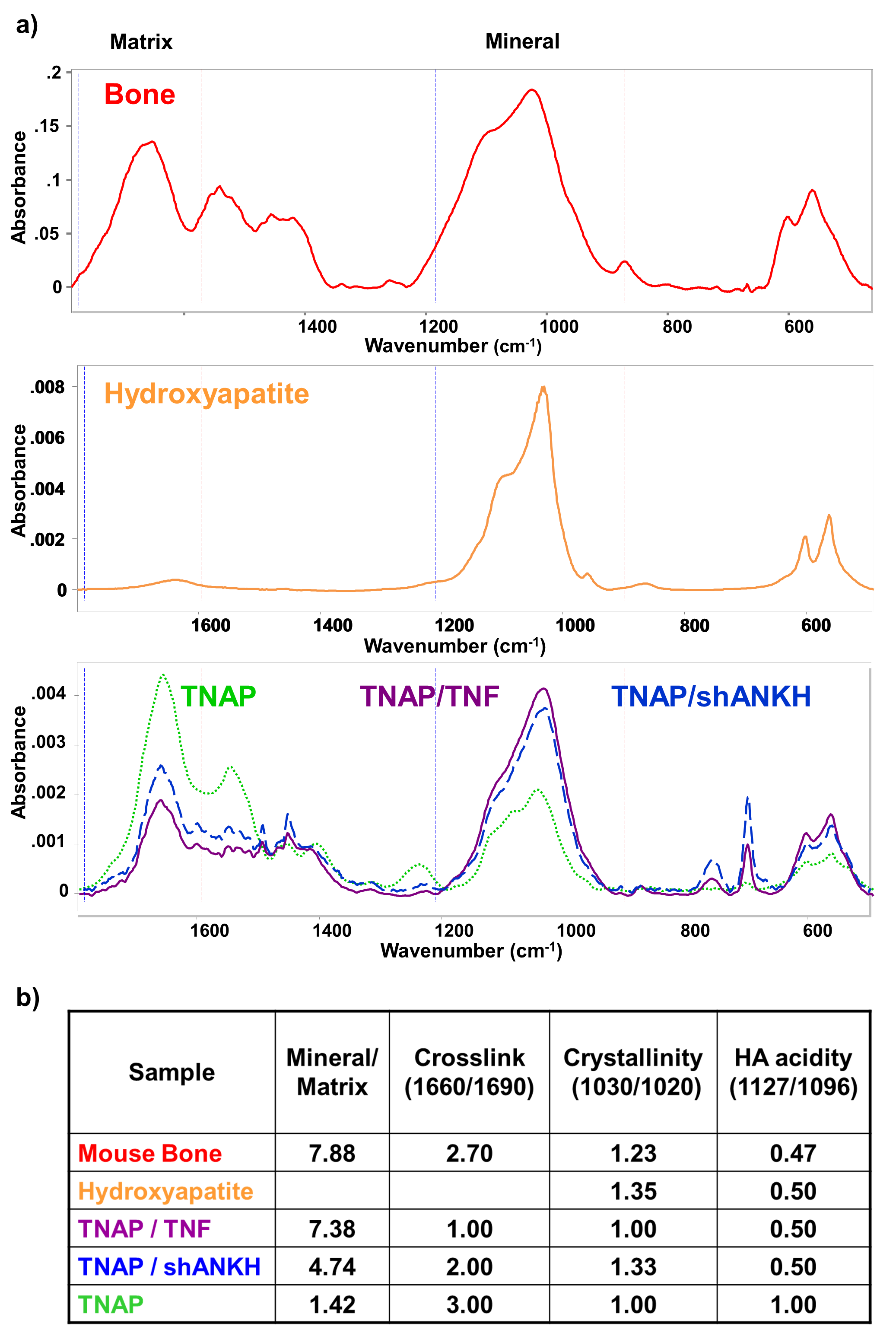


Supplementary Figure 2. Deposition of Hydroxyapatite by transduced bNP cells. a) FTIR spectra of mouse bone, Hydroxyapatite and mineral deposited by TNAP (dotted, green line), TNAP/shANKH (dashed, blue line) and TNAP/TNF (solid, purple line) transduced bNPs. b) Mineral composition was assessed by examining the magnitude of the area of the amide 1 peak (1710-1590), mineral peak (1215-900), carbonate peak (852-890), HA crystallinity peak (500-670), and the crosslink intensity (1660/1690 ratio), crystallinity intensity (1030/1020 ratio), and acid phosphate (1128/1096 ratio).


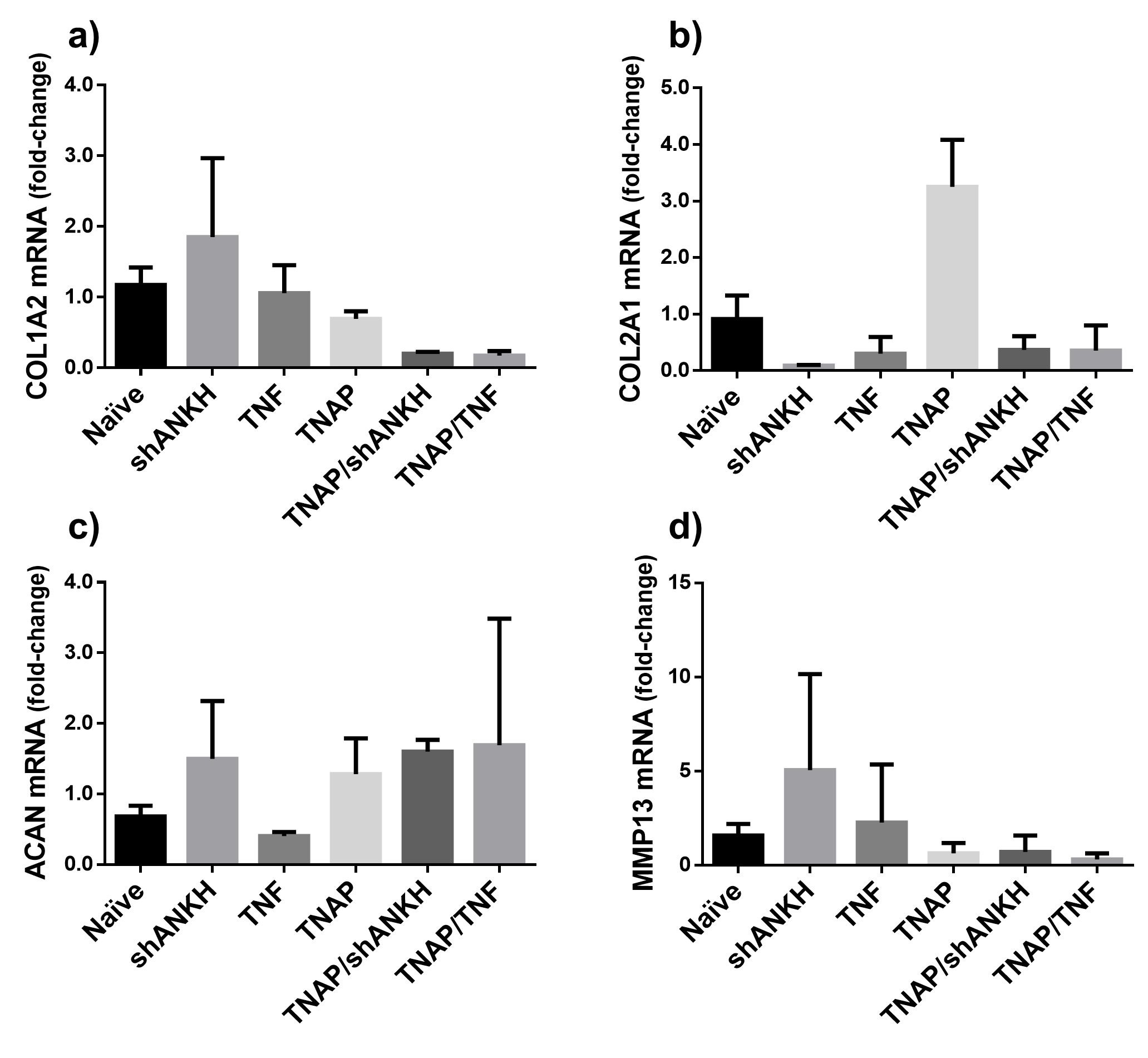


Supplementary Figure 3. Genes expression of a) COL1A2; b) COL2A1; c) ACAN; d) MMP13 gene expression in transduced bNPs. (n=3). Data analyzed using One-way ANOVA.


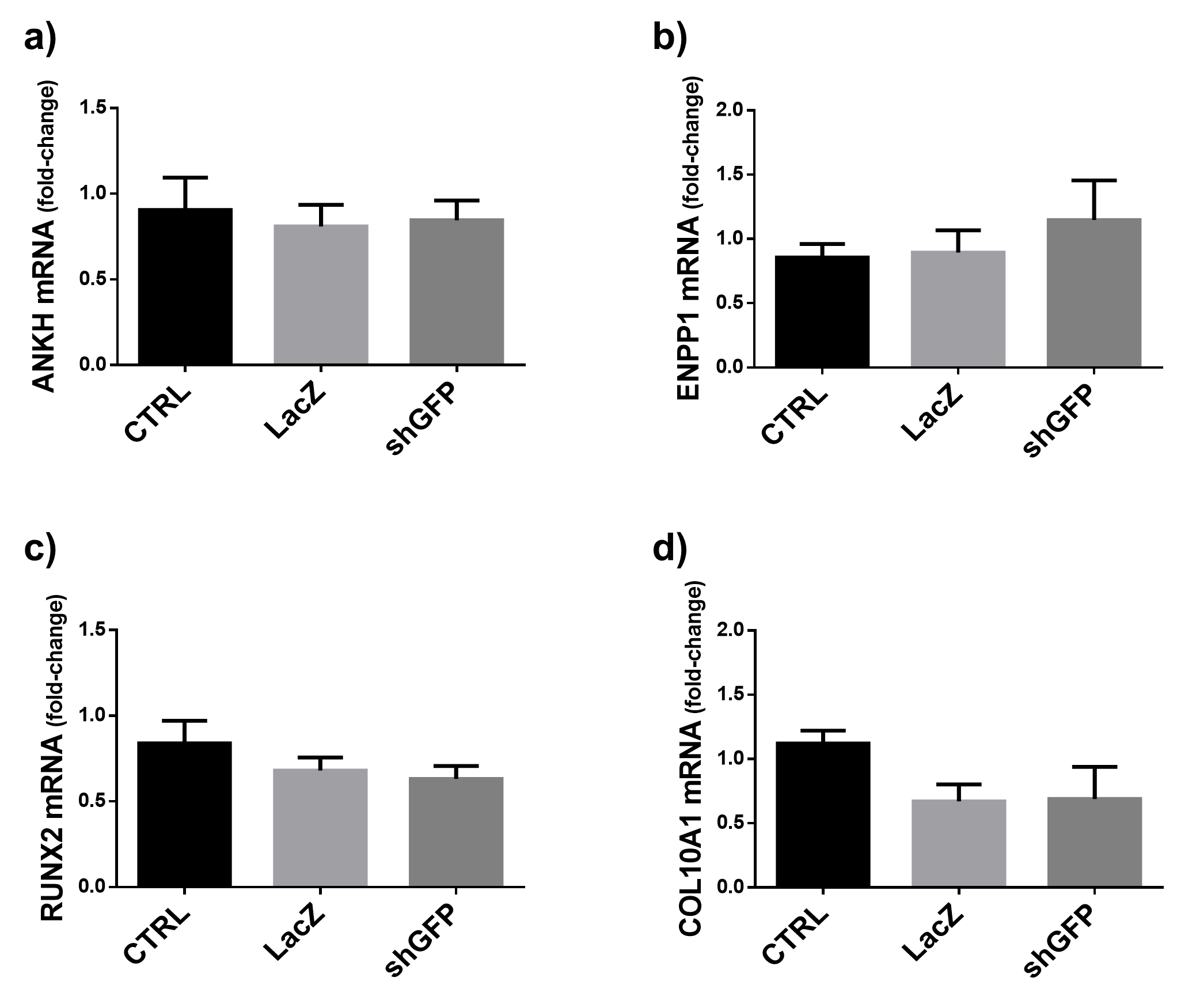


Supplementary Figure 4. No changes in expression of a) ANKH, b) ENPP1, c) RUNX2, d) COL10A in naïve, LacZ and shGFP transduced bNP cells (n=3). No significant differences were found between group. Data analyzed using One-way ANOVA.

a) b)

Supplementary Figure 5. Collagen X immunostaining affected by TNAP/ANKH/ENPP1 relative expression status. Pellet cultures of bNP cells either (a) naïve or (b) gene-programmed to express TNAP & TNF were immunostained with colX / counterstained with DAPI, were digitally quantified for colX immunofluorescence intensity, and showed increased colX expression in the TNAP/TNF condition (p<0.001, data analyzed using Student’s t-test).


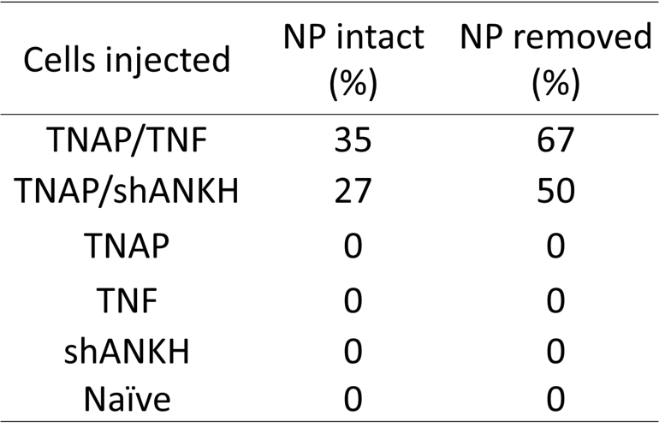


Supplementary Figure 6. Percentage of discs with an increase of BV/TV. Mineral content in discs injected with naïve cells was used as a base line (5-fold increase was observed in some of the discs in this group); discs were increase of BV/TV was higher than 7-fold were considered as a positive outcome of injection.
